# Mental health disorders among palestinians: a systematic review of prevalence, risk factors, and barriers to care

**DOI:** 10.1192/j.eurpsy.2026.11489

**Published:** 2026-07-31

**Authors:** F. Zaouali, I. Anes

**Affiliations:** 1 Emergency department and mobile emergency and resuscitation service, Tahar Sfar University Hospital, Mahdia; 2 Outpatient psychiatric consultation, Hadj Ali Soua Regional Hospital, Ksar Hellal, Monastir, Tunisia

## Abstract

**Introduction:**

The protracted conflict and repeated displacement in Palestine have subjected the population to extensive psychosocial stressors. Mental health disorders, including post-traumatic stress disorder (PTSD), depression, and anxiety, are reported at high rates, but a comprehensive synthesis of this data is lacking.

**Objectives:**

This study aims to systematically review the literature on the prevalence of mental health disorders among Palestinians, identify key risk factors, and examine barriers to accessing mental health care.

**Methods:**

A systematic search was conducted in PubMed, Scopus, PsycINFO, and Google Scholar for studies published between 2000 and 2025. Keywords included “Palestine,” “Gaza,” “West Bank,” “mental health,” “depression,” “PTSD,” “anxiety,” “refugees,” and “displaced persons.” Inclusion criteria comprised quantitative studies reporting prevalence or risk factors for mental disorders among Palestinians. Data on sample size, assessment tools, prevalence rates, and care barriers were extracted. Study quality was assessed using the Newcastle-Ottawa Scale adapted for cross-sectional studies.

**Results:**

Thirty studies with sample sizes ranging from 150 to 2,500 participants were included. PTSD prevalence ranged from 30% to 68%, while depression affected 35% to 70%, with highest rates in Gaza. Anxiety disorders were reported in 25% to 55% of participants. Female gender, exposure to violence, displacement, and socioeconomic deprivation were consistently linked to increased risk. Barriers to care included stigma, limited resources, and accessibility challenges, particularly in refugee camps.

**Conclusions:**

Palestinians living under ongoing conflict exhibit a high burden of mental health disorders exacerbated by socio-political and economic hardships. Effective mental health interventions must be culturally sensitive and address access barriers. Further longitudinal and intervention research is needed to guide policies and improve mental health outcomes.

**Disclosure of Interest:**

None Declared

